# Gender differences in risk exposures for acute hepatitis C infection in Taiwan: a nationwide case–control study

**DOI:** 10.1186/s12889-023-14995-3

**Published:** 2023-01-12

**Authors:** Hsin-I Huang, Chia-Ping Su, Wan-Ting Huang, Wan-Chin Chen

**Affiliations:** 1 Center for Research, Diagnostics and Vaccine Development, Taiwan Centers for Disease Control, No. 161, Kunyang St., Nangang Dist., Taipei City, 115 Taiwan; 2 Preventive Medicine Office, Taiwan Centers for Disease Control, No. 6, Linsen S. Rd., Zhongzheng Dist., Taipei City 100, Taiwan

**Keywords:** Hepatitis C, Healthcare-associated risk exposures, Gender difference

## Abstract

**Background:**

In Taiwan, medical providers are required to report all acute hepatitis C (AHC) patients to National Notifiable Disease Surveillance System (NNDSS). Identifying factors associated with AHC may inform the strategies to prevent the spread of hepatitis C virus (HCV). We used the national surveillance data to assess gender difference in risk factors associated with AHC in Taiwan and propose control measures in at-risk groups.

**Methods:**

We conducted a nationwide case–control study using data from NNDSS and AHC case investigation questionnaires, for the period of March 6, 2014–December 31, 2016. Cases were AHC confirmed in NNDSS; controls were reported AHC with negative HCV nucleic acid test and negative serum anti-HCV antibody. We used bivariate analysis to identify characteristics and risk exposures for AHC and conducted gender stratified analyses.

**Results:**

We identified 602 AHC cases (66.9% males, median age 48 years) and 90 controls. Older age, male gender (OR: 1.85, 95% CI: 1.18–2.90), history of viral hepatitis (OR: 7.93, 95% CI:1.91–32.88), history of sexually transmitted infections (OR: 21.02, 95% CI: 2.90–152.43), and having healthcare-associated risk exposures (OR: 2.02, 95% CI: 1.25–3.25) were associated with AHC. Stratified analyses showed receiving intravenous infusion, history of hepatitis B, syphilis, and human immunodeficiency virus infection were risk factors for male AHC; receiving hemodialysis was risk factor for females.

**Conclusions:**

Our study demonstrates risk factors for AHC in Taiwan with gender difference. Proper infection control practices in healthcare settings and interventions targeting male patients with HIV and other STIs, remain crucial to prevent individuals from AHC.

## Introduction

Hepatitis C is a liver disease caused by the bloodborne hepatitis C virus (HCV). HCV is highly transmissible through percutaneous exposure to blood, for example, drug injection using medical device with inadequate sterilization, unscreened blood and blood product transfusion, and condomless sex [1, 2]. Persistent infection and chronic hepatitis are hallmarks of HCV infection, which may lead to cirrhosis and hepatocellular carcinoma with increasing healthcare costs and mortality if left untreated [3].

Recent study estimates global seroprevalence is 2.5% with 177.5 million adults infected with HCV [4]. The seroprevalence of anti-HCV in Taiwan is around 4%–5% which was higher than estimated global seroprevalence [3, 5]. In Taiwan, studies during 1990–2001 stated that there were great geographical variations in the seroprevalence of HCV, and the major route of transmission was injection drug use with shared syringes or other paraphernalia. Individual risk factors included older age, female gender, and blood transfusion [6–11]. Studies after 2012 indicated that HCV transmission occurs among persons living with human immunodeficiency virus (HIV), especially men who have sex with men (MSM), as a result of sexual contact with an HCV-infected partner [12, 13]. Syringe sharing and incarceration were associated with HCV/HIV co-infection or HCV mono-infection among people who inject drugs (PWID) [14]. These recent studies, however, mainly enrolled specific groups such as MSM, HIV patients or PWID, rather than the general population as nationally representative studies. In particular, gender difference in risk exposures of HCV are less studied.

With improving healthcare resources and infection control at healthcare facilities, the major transmission routes and risk factors for acute hepatitis C (AHC) in Taiwan can change over the past decades. As the development and use of direct-acting antivirals has substantially improved HCV treatment, the World Health Organization sets a target for the elimination of HCV as a public health concern by 2030 [15]. Identifying factors associated with AHC may inform the strategies to prevent the spread of HCV. We conducted a nationwide case–control study using the national surveillance data to assess gender difference in risk factors associated with AHC in Taiwan and propose control measures in at-risk groups.

## Materials and methods

### AHC surveillance in Taiwan

Taiwan Centers for Disease Control (TCDC) uses the National Notifiable Disease Surveillance System (NNDSS) to monitor AHC since 1999 to identify the risk groups and transmission routes, and to control HCV transmission. All medical providers are required to report to web-based NNDSS if patients fulfilled the reporting criteria for AHC within 7 days of diagnoses.

In Taiwan, various surveillance case definitions for AHC have been used since 1999 [13]. At the time of this study, the reporting criteria, established on March 6, 2014, included clinical criteria (having all of the followings: symptoms of acute hepatitis or a serum alanine aminotransferase level ≥ 100 IU/L; a positive anti-HCV antibody; and exclusion of acute exacerbation of chronic hepatitis or hepatitis of other etiologies) and laboratory criteria (any of the following: anti-HCV antibody seroconversion within one year; or a positive serum HCV nucleic acid test (NAT) with a negative anti-HCV antibody at the same time). A confirmed AHC patient is a patient that meets either the clinical or laboratory criteria, or both. According to AHC Disease Control Manual in Taiwan, public health professionals were required to conduct an investigation for reported AHC cases using a standardized questionnaire. The questionnaire collects information on demographics, medical histories, household or close contacts, and risk behaviors or receipt of invasive procedures in the past six months. These data are then entered into the Central Investigation System (CIS), which is also operated by TCDC, within 30 days of patient confirmation.

### Case–control study

We conducted a nationwide, retrospective case–control study using data from NNDSS and CIS. The study population included all reported AHC patients during March 6, 2014 to December 31, 2016. We defined AHC cases as reported patients confirmed by NNDSS. Controls were reported AHC patients who did not meet the AHC confirmation criteria, and had negative anti-HCV antibody and negative HCV NAT in the NNDSS database. The attributes of interest included age, gender, residential area, history of hepatitis or sexually transmitted infections (STIs), and sexual and potential parenteral exposures 6 months prior illness onset. We defined “metropolitan area” as the jurisdiction of cities of Taipei, New Taipei, Taoyuan, Taichung, Tainan, and Kaohsiung; “history of hepatitis” as ever had viral hepatitis A, B, C, D or E; “healthcare-associated exposure” as parenteral exposure which related to healthcare delivery in inpatient, outpatient, and long-term care facilities in recent 6 months, including intravenous infusions, dental procedures, surgery, blood transfusion, hemodialysis, or transplant; “STIs” as syphilis, gonorrhea, *Condyloma accuminatum*, genital *Chlamydia trachomatis*, genital rash, and HIV.

### Data analyses

The data were analyzed using the Epi Info™ program, version 7.2.2.6. We used bivariate analysis and compared the proportion of attributes and potential risk exposures between cases and controls by using chi-square or Fisher’s exact tests. The relationship between age and AHC was assessed using the Student’s t test or Mann–Whitney test. All comparisons were two-tailed and a *p*-value < 0.05 was considered significant. The odds ratios (OR) with 95% confidence interval (95% CI) were calculated overall and stratified by gender (male and female).

## Results

**Fig. 1:**
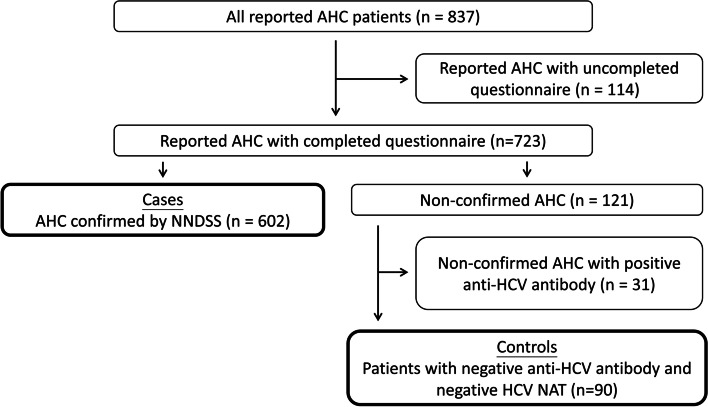
Flow diagram of AHC cases and controls, Taiwan, March 6, 2014–December 31, 2016 Abbreviations: AHC: acute hepatitis C; HCV: hepatitis C virus; NAT, nucleic acid test

Total 837 AHC were reported to NNDSS during March 6, 2014–December 31, 2016. Of the 723 reported AHC patients whose investigation questionnaires were completed, 602 were confirmed by NNDSS (cases) and 90 had negative anti-HCV antibody and negative HCV NAT (controls) (Fig. 1).

### Patient characteristics and risk exposures

**Fig. 2:**
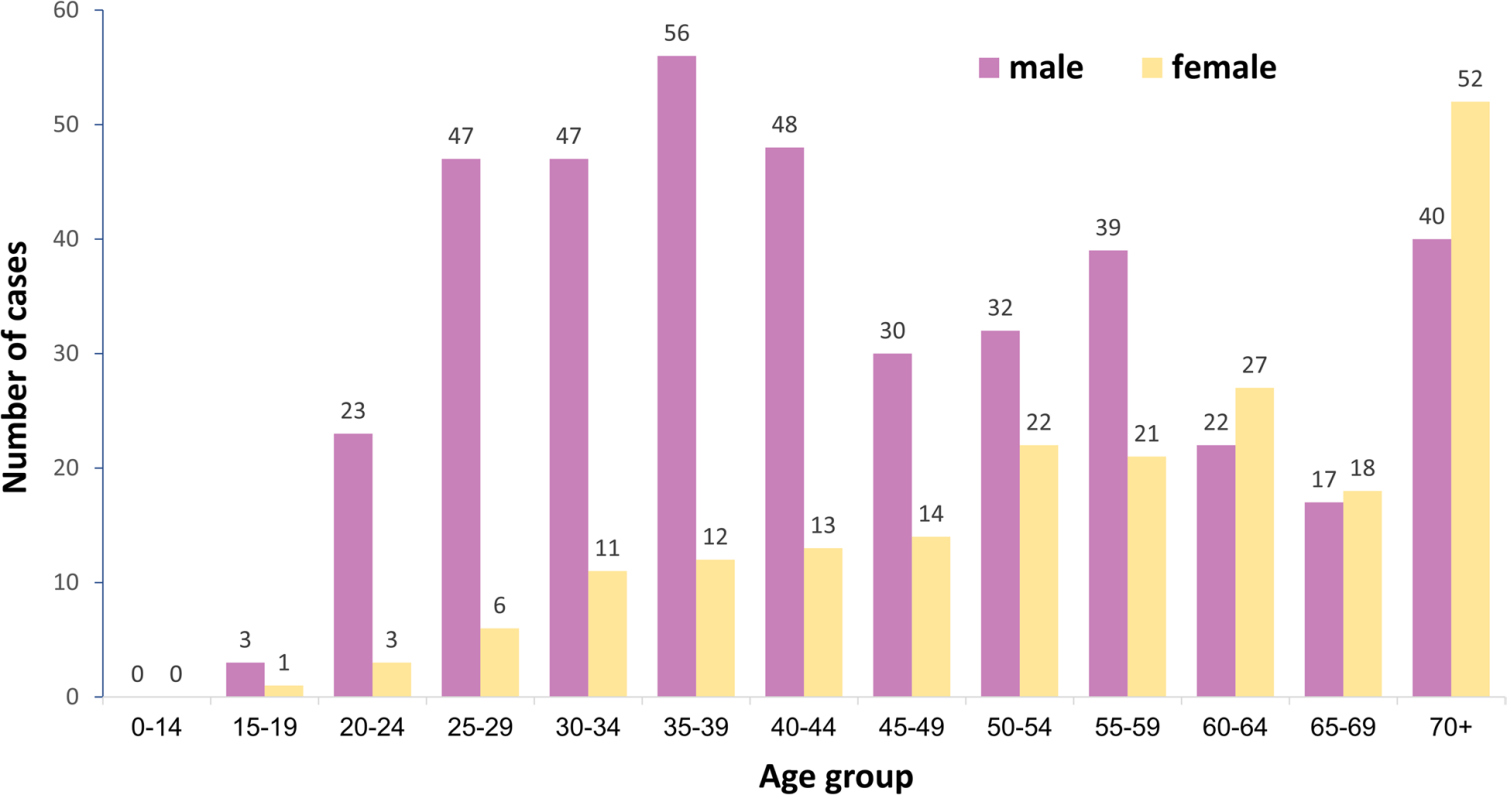
Confirmed acute hepatitis C cases by age group and gender, Taiwan, March 2014–December 2016 (*n* = 602)

Of 602 AHC cases, 403 (66.9%) were males and median age was 48 years (range, 17–96). Age and gender distribution of the cases were shown in Fig. 2; 257 (42%) of the cases were male under 50 years old.

Patient characteristics and risk exposures were shown in Table 1. Among AHC cases, 279 (46.4%) had recent healthcare-associated risk exposures; 115 (19.1%) had history of STIs while only one in the controls (*p* < 0.05). Older age (*p* < 0.01), male gender (OR: 1.85, 95% CI: 1.18–2.90), history of viral hepatitis A, B, or C (OR: 7.93, 95% CI: 1.91–32.88), having healthcare-associated risk exposures (OR: 2.02, 95% CI: 1.25–3.25), and receiving intravenous infusion in recent 6 months (OR: 2.51, 95% CI: 1.30–4.84) were associated with overall acute HCV infection. History of hepatitis B (*n* = 58, 9.6%), syphilis (*n* = 52, 8.6%), HIV (*n* = 82, 13.6%), and hemodialysis (*n* = 39, 6.5%) were reported in AHC cases but not in controls (all were *p* < 0.05).

**Table 1:** Characteristics and risk exposures of acute hepatitis C cases and controls, Taiwan, March 2014–December 2016

Characteristics and risk exposures	Cases (*n* = 602)	Controls (*n* = 90)	OR (95% CI)
Age, years, median (range)	48 (17–96)	32 (17–63)	*–* ^a^
Gender, male, n (%)	403 (66.9)	47 (52.2)	1.85 (1.18–2.90)
Residents in metropolitan area, n (%)	385 (64.0)	67 (74.4)	0.61 (0.37–1.01)
History of viral hepatitis, n (%)	81 (16.2)	2 (2.4)	7.93 (1.91–32.88)
Hepatitis A	4 (0.7)	0 (0)	undefined
Hepatitis B	58 (9.6)	0 (0)	undefined^b^
Hepatitis C	24 (4.0)	2 (2.2)	1.83 (0.42–7.87)
Hepatitis D	0 (0)	0 (0)	undefined
Hepatitis E	0 (0)	0 (0)	undefined
History of STIs, n (%)	115 (19.1)	1 (1.1)	21.02 (2.90–152.43)^b^
Syphilis	52 (8.6)	0 (0)	undefined^b^
Gonorrhea	10 (1.7)	0 (0)	undefined
*Condyloma accuminatum*	8 (1.3)	0 (0)	undefined
Genital *Chlamydia trachomatis*	0 (0)	0 (0)	undefined
Genital rash	1 (0.2)	0 (0)	undefined
HIV	82 (13.6)	0 (0)	undefined^b^
Risk exposures in recent 6 months, n (%)	398 (66.1)	52 (57.8)	1.43 (0.91–2.24)
Condomless sex	110 (18.3)	19 (21.1)	0.84 (0.48–1.44)
Healthcare-associated risk exposures	279 (46.4)	27 (30.0)	2.02 (1.25–3.25)
Intravenous infusions	156 (25.9)	11 (12.2)	2.51 (1.30–4.84)
Dental procedures	86 (14.3)	13 (14.4)	0.99 (0.53–1.85)
Surgery	47 (7.8)	2 (2.2)	3.73 (0.89–15.62)
Blood transfusion	38 (6.3)	2 (2.2)	2.96 (0.70–12.51)
Hemodialysis	39 (6.5)	0 (0)	undefined^b^
Transplant	2 (0.3)	0 (0)	undefined
Acupuncture	28 (4.7)	4 (4.4)	1.05 (0.36–3.06)
Sharing razors or tooth brush	16 (2.7)	2 (12.2)	1.20 (0.27–5.31)
Piercing	15 (2.5)	2 (2.2)	1.12 (0.25–5.00)
People who injected drugs	13 (2.2)	0 (0)	undefined
Sharing needles or other paraphernalia	4 (0.7)	0 (0)	undefined
Occupational needle stick injuries	10 (1.7)	2 (2.2)	0.74 (0.16–3.45)
Non-occupational needle stick injuries	0 (0)	0 (0)	undefined

*Abbreviations*: *OR* odds ratio, *CI* confidence interval, *STI* sexually transmitted infection, *HIV* human immunodeficiency virus

^a^*p* < 0.01 by Mann–Whitney test

^b^*p* < 0.05 by Fisher’s exact test

### Gender stratified analyses

Table 2 summarized patient characteristics and risk exposures by gender. Compared with the controls, older age (*p* < 0.01), history of viral hepatitis A, B, and C (OR: 4.58, 95% CI: 1.08–19.4), having healthcare-associated risk exposures (OR: 2.43, 95% CI: 1.18–5.04), and ever receiving intravenous infusions in healthcare facilities in recent 6 months (OR: 6.38, 95% CI: 1.52–26.80) were associated with AHC in males. History of STIs (*n* = 112, 27.8%) while only one in the controls, history of hepatitis B (*n* = 46, 11.4%), syphilis (*n* = 52, 12.9%), and HIV (*n* = 82, 20.4%) while none in controls were reported in male AHC cases (all were *p* < 0.01). Three of the four cases with history of hepatitis A reported having condomless sex with male partners.

**Table 2:** Characteristics and risk exposures of acute hepatitis C cases and controls by gender, Taiwan, March 2014–December 2016

Characteristics and risk exposures		Male			Female	
	Cases (*n* = 403)	Controls (*n* = 47)	OR (95% CI)	Cases (*n* = 199)	Controls (*n* = 43)	OR (95% CI)
Age, years, median (range)	42 (17–94)	32 (18–63)	–^a^	59 (19–96)	32 (17–63)	–^a^
Residents in metropolitan area, n (%)	266 (66.0)	35 (74.5)	0.67 (0.33–1.32)	119 (59.8)	32 (74.4)	0.51 (0.24–1.07)
History of viral hepatitis, n (%)	61 (17.9)	2 (4.6)	4.58 (1.08–19.41)	20 (12.6)	0 (0)	undefined^b^
Hepatitis A	4 (1.0)	0 (0)	undefined	0 (0)	0 (0)	undefined
Hepatitis B	46 (11.4)	0 (0)	undefined^b^	12 (6.0)	0 (0)	undefined
Hepatitis C	15 (3.7)	2 (4.3)	0.87 (0.19–3.93)	9 (4.5)	0 (0)	undefined
Hepatitis D	0 (0)	0 (0)	undefined	0 (0)	0 (0)	undefined
Hepatitis E	0 (0)	0 (0)	undefined	0 (0)	0 (0)	undefined
History of STIs, n (%)	112 (27.8)	1 (2.1)	17.70 (2.41–129.93)	3 (1.5)	0 (0)	undefined
Syphilis	52 (12.9)	0 (0)	undefined^b^	0 (0)	0 (0)	undefined
Gonorrhea	10 (2.5)	0 (0)	undefined	0 (0)	0 (0)	undefined
*Condyloma accuminatum*	7 (1.7)	0 (0)	undefined	1 (0.5)	0 (0)	undefined
Genital Chlamydia trachomatis	0 (0)	0 (0)	undefined	0 (0)	0 (0)	undefined
Genital rash	1 (0.3)	0 (0)	undefined	0 (0)	0 (0)	undefined
HIV	82 (20.4)	0 (0)	undefined^b^	0 (0)	0 (0)	undefined
Risk exposures in recent 6 months, n (%)	260 (64.5)	28 (59.6)	1.23 (0.67–2.29)	138 (69.5)	24 (55.8)	1.79 (0.91–3.51)
Condomless sex	91 (22.6)	11 (23.4)	0.95 (0.47–1.95)	19 (9.6)	8 (18.6)	0.46 (0.19–1.14)
Healthcare-associated risk exposures	160 (39.7)	10 (21.3)	2.43 (1.18–5.04)	119 (59.8)	17 (39.5)	2.28 (1.16–4.46)
Intravenous infusions	89 (22.1)	2 (4.3)	6.38 (1.52–26.80)	67 (33.7)	9 (21.0)	1.92 (0.87–4.23)
Dental procedures	49 (12.2)	7 (14.9)	0.79 (0.34–1.86)	37 (18.6)	6 (14.0)	1.41 (0.55–3.58)
Surgery	24 (6.0)	1 (2.1)	2.91 (0.39–22.04)	23 (11.6)	1 (2.3)	5.49 (0.72–41.80)
Blood transfusion	18 (4.5)	0 (0)	undefined	20 (10.1)	2 (4.7)	2.29 (0.51–10.19)
Hemodialysis	22 (5.5)	0 (0)	undefined	17 (8.5)	0 (0)	undefined^b^
Transplant	1 (0.3)	0 (0)	undefined	1 (0.5)	0 (0)	undefined
Acupuncture	17 (4.2)	2 (4.3)	0.99 (0.22–4.43)	11 (5.5)	2 (4.7)	1.20 (0.26–5.62)
Sharing razors or tooth brush	16 (4.0)	2 (4.3)	0.93 (0.21–4.18)	0 (0)	0 (0)	undefined
Piercing	11 (2.7)	1 (2.1)	1.29 (0.16–10.22)	4 (2.0)	1 (2.3)	0.86 (0.09–7.91)
People who inject drugs	9 (2.2)	0 (0)	undefined	4 (2.0)	0 (0)	undefined
Sharing needles or other paraphernalia	3 (0.7)	0 (0)	undefined	1 (0.5)	0 (0)	undefined
Occupational needle stick injuries	7 (1.7)	0 (0)	undefined	3 (1.5)	2 (4.7)	0.31 (0.05–1.94)
Non-occupational needle stick injuries	0 (0)	0 (0)	undefined	0 (0)	0 (0)	undefined

*Abbreviations*: *OR* odds ratio, *CI* confidence interval, *STI* sexually transmitted infection, *HIV* human immunodeficiency virus

^a^*p* < 0.01 by Mann–Whitney test

^b^*p* < 0.05 by Fisher’s exact test

The age of female AHC cases (median 59 years, range 19–96) was significant older than that of male cases (median 42 years, range 17–94) (*p* < 0.01) (Table 2). In females, AHC was associated with older age (*p* < 0.01) and having healthcare-associated exposures (OR: 2.28, 95% CI: 1.16–4.46). History of viral hepatitis A, B, and C (*n* = 20, 12.6%) (*p* = 0.02) and receiving hemodialysis in recent 6 months (*n* = 17, 8.5%) (*p* = 0.048) were reported in female AHC cases but none in controls.

## Discussion

Our study showed that healthcare-associated exposure was the most common exposure among AHC cases in Taiwan (279/602, 46.4%). Risk factors for AHC in Taiwan included older age, male gender, history of viral hepatitis A, B, or C, history of STIs, receiving intravenous infusion in recent 6 months, and hemodialysis. We also observed gender difference: male cases were younger than female cases; and history of STIs and receiving recent intravenous infusions were identified as AHC risk exposures among males, compared with hemodialysis identified among females.

Findings from a 1991–1992 study had proposed that female gender, older age, history of blood transfusion and geographical variation by limited healthcare resources were associated with HCV infection in Taiwan [11]; however, none of these risk factors except older age was established in our study. This difference may be attributed to the introduction of several measures, including universal healthcare through National Health Insurance since 1995, use of disposable medical devices since 1980, anti-HCV antibody screening for all blood donors since 1992, and adopting HCV nucleic acid amplification technology in Taiwan Blood Services Foundation since 2013 [16].

In high-income countries, healthcare associated exposures are a recognized transmission route in HCV infection, as shown by reports in several European countries and the US [17]. In most of the occurrences, the putative mechanism of infection was patient-to-patient transmission because healthcare personnel failed to adhere to fundamental principles of infection control and aseptic technique (for example, reuse of syringes or lancing devices) [2, 18–20]. The prevalence of HCV infection is historically high in hemodialysis units due to environmental contamination by HCV-positive blood and insufficient adherence to hygienic measures [17, 21]. Our study also observed that receiving intravenous infusion and hemodialysis was associated with male and female AHC, respectively.

In Europe, the majority of HCV cases were aged between 25 and 44 years in 2016 [22]; in the US, the median age of AHC patients was 31 years with 46.7% reported as PWID in 1982–2006, and patients aged between 20 and 29 years had the highest HCV incidence in 2001–2016 [23, 24]. Findings from our study indicated that AHC patients in Taiwan were older than those in western countries; by contrast, PWID, was not considered a major risk factor for AHC in Taiwan according to our study. In addition, persons with higher age are expected to have more parenteral, healthcare-associated exposures, and more likely to receive liver function or HCV tests due to higher healthcare needs and utilization. Retrospective identification of specific infection control breaches in healthcare facilities, however, was difficult because most of AHC infection were asymptomatic; the incubation period ranged 14–180 days, and multiple parenteral exposures could occur in the elderly patients [17].

In accordance with recent findings from studies in the US and Europe, our results suggested that more than half of the AHC cases were men [22, 24]. The higher proportion of syphilis in male AHC cases than controls supports a contributing role of syphilitic ulcers to HCV acquisition through impairing mucosal integrity [13, 25]. Several studies also reveal that past or recent syphilis was associated with HCV seroconversion among MSM or HIV patients [26, 27]. In this study, most of the AHC cases with a history of viral hepatitis reported hepatitis B (58/81, 71.6%) and a minority (4/81, 0.9%, all males) reported hepatitis A. Hepatitis B infections share similar transmission route with HCV, and during the study period, a hepatitis A outbreak emerged in June 2015 which mainly affected MSM and patients with HIV or other STIs including syphilis with possible transmission through sexual contacts or at MSM events [28]. Three of the four male AHC cases with history of hepatitis A in our study also reported having condomless sex with male partners.

Our study had several limitations. Because most infections with HCV are asymptomatic, under-reporting, particularly in patients with less needs and access to healthcare, can occur. Second, it could be difficult for physicians to differentiate patients with AHC from chronic hepatitis C among those did not have previous diagnosis of HCV; reported and misclassified as AHC was possible in the AHC cases. Third, we selected from NNDSS the reported but non-confirmed AHC patients with negative anti-HCV and negative HCV NAT as controls; however, a relatively small number of controls were available. These controls may be biased and not representative of the general non-AHC population. This report was based on public health surveillance data to identify possible risk groups in Taiwan, a further cohort or hospitalized-based case–control study would be needed to confirm the cause of acute hepatitis C transmission. Lastly, in Taiwan, personal information on the number/gender of sexual partners and history of STIs are considered sensitive; use of control substances such as opioid or methamphetamine was against the criminal law and may lead to years of incarceration. Patients may tend to hold back this information during the interview and we may underestimate condomless sex, STIs, and PWID both in cases and controls.

## Conclusions

This nationwide case–control study identified older age, history of viral hepatitis A, B, or C, and healthcare-associated exposures as risk factors for AHC in Taiwan during the period of 2014–2016. Receiving intravenous infusion, history of hepatitis B, syphilis, and HIV were risk factors for males, while receiving hemodialysis was risk factor for females.

We recommend establishment of a multifaceted framework to investigate possible healthcare-associated AHC; provide training to healthcare professionals; implement and maintain appropriate infection control practices and techniques, and improve oversight. We also recommend HCV testing and interventions targeting male patients with HIV and other STIs to reduce AHC.

## Data Availability

The data that support the findings are available from NNDSS and CIS and the containing information could compromise the privacy of the reported patients. Restrictions apply to the availability of these data, which were used under license for the current study, and so are not publicly available. Data are however available from the corresponding author upon reasonable request and the permission of TCDC.
